# Ultrasound halo count in the differential diagnosis of atherosclerosis and large vessel giant cell arteritis

**DOI:** 10.1186/s13075-023-03002-0

**Published:** 2023-02-14

**Authors:** Irene Monjo-Henry, Elisa Fernández-Fernández, José María Mostaza, Carlos Lahoz, Juan Molina-Collada, Eugenio de Miguel

**Affiliations:** 1 Rheumatology Department, Hospital Universitario La Paz, Madrid, Spain; 2 Department of Internal Medicine, Hospital Carlos III, Madrid, Spain; 3 Rheumatology Department, Hospital General Universitario Gregorio Marañón, Madrid, Spain

**Keywords:** Giant cell arteritis, Large vessel vasculitis, Atherosclerosis, Diagnosis, Imaging, Ultrasonography

## Abstract

**Objective:**

To determine the diagnostic discriminant validity between large vessel giant cell arteritis (LV-GCA) and atherosclerosis using ultrasound (US) intima-media thickness (IMT) measurements.

**Methods:**

We included 44 patients with LV-GCA and 42 with high-risk atherosclerosis. US examinations of the axillary, subclavian, and common carotid arteries (CCA) were systematically performed using a MylabX8 system (Genoa, Italy) with a 4–15-MHz probe. IMT ≥ 1 mm was accepted as pathological.

**Results:**

The LV-GCA cohort included 24 females and 20 males with a mean age of 72.8 ± 7.6 years. The atherosclerosis group included 25 males and 17 females with a mean age of 70.8 ± 6.5 years. The mean IMT values of all arteries included were significantly higher in LV-GCA than in atherosclerosis. Among LV-GCA patients, IMT ≥ 1 mm was seen in 31 axillary, 30 subclavian, and 28 CCA. In the atherosclerotic cohort, 17 (38.6%) had IMT ≥ 1 mm with axillary involvement in 2 patients, subclavian in 3 patients, carotid distal in 14 patients (5 bilateral), and isolated carotid proximal affectation in 1 case. A cutoff point greater than 1 pathological vessel in the summative count of axillary and subclavian arteries or at least 3 vessels in the count of six vessels, including CCA, showed a precision upper 95% for GCA diagnosis.

**Conclusion:**

The IMT is higher in LV-GCA than in atherosclerosis. The proposed US halo count achieves an accuracy of > 95% for the differential diagnosis between LV-GCA and atherosclerosis. The axillary and subclavian arteries have higher discriminatory power, while carotid involvement is less specific in the differential diagnosis.

## Introduction

Giant cell arteritis (GCA) is the most common vasculitis in the elderly [1–3]. We can differentiate two patterns of vascular involvement in GCA: the classic cranial pattern, with headache and ischemic symptoms, and the extra-cranial large vessel pattern (LV-GCA), which presents with constitutional symptoms such as fever, asthenia, and polymyalgia rheumatica. The focus of study in GCA has traditionally been on temporal arteries. But there is growing evidence that LV involvement is more frequent than previously thought. The aorta, axillary, and other large vessels (i.e., extra-cranial arteries) can be affected in more than 50% of GCA cases [4–7].

For the diagnosis of GCA, temporal artery biopsy has long been regarded as the gold standard, but given the patched character of this vasculitis, there can be a false-negative rate of up to 40% [8]. In addition, temporal artery biopsy is not useful for diagnosing LV involvement. Thus, imaging is the cornerstone of LV-GCA diagnosis [9].

LV-GCA patients have a higher risk of vascular complications such as aortic dilation [10], and these appear to be predictors of recurrence and require longer treatment [11]. Therefore, it is important to detect this subgroup of patients. Such is the importance of imaging in large vessel vasculitis (LVV) that in 2018, the European Alliance of Associations for Rheumatology (EULAR) published the first recommendations for the use of imaging in the diagnosis and monitoring of LVV. Color Doppler sonography (CDS) was the first established imaging test to be performed in patients with suspected GCA. In patients with high clinical suspicion of GCA and a positive imaging test, the diagnosis can be performed without additional testing [9]. Moreover, we know that atherosclerosis, a frequent pathology in the age group of typical patients with GCA, is associated with increases in IMT [12, 13] and potentially indicates a problem in the differential diagnosis between both diseases.

The Outcome Measures in Rheumatology Clinical Trials (OMERACT) ultrasound (US) subgroup on LVV provided definitions of the normal US appearance and key elementary lesions of vasculitis of the temporal arteries and LV based on international expert consensus. They defined a normal extra-cranial large artery as a “pulsating hardly compressible artery with anechoic lumen.” The intima-media complex was defined as “homogenous hypoechoic or anechoic echo-structure delineated by two parallel hyperechoic margins (‘double line pattern’) which is surrounded by mid-echoic to hyperechoic tissue” and the halo sign in cranial or LVV as “homogenous, hypoechoic wall thickening, well delineated towards the luminal side, visible both in longitudinal and transverse planes; it is most commonly concentric in transverse scans” [14]. However, this raises a new, unanswered question: how do we determine which arteries should be assessed? Additionally, what is the cutoff point for accurately differentiating between a normal and pathological intima-media thickness (IMT) in the arterial wall? To date, a cutoff measure of 0.9 to 1.5 mm has been used in different studies, but there is no consensus or evidence for the selection of a specific value [5, 15–17].

Our main objective was to assess the discriminant validity of the US halo/IMT to differentiate between LV-GCA and atherosclerosis.

## Materials and methods

### Patients

This study included two different groups of patients. Group 1 was integrated by the consecutive enrolment of those patients at our GCA fast-track clinic (April 2017 to July 2020) who had a LV-GCA diagnosis. Patients needed to present an US halo sign consistent with GCA in at least one of the arteries explored and have manifestations consistent with GCA. The clinical diagnosis of the doctor in charge, which had to be confirmed after at least 1 year of follow-up, was used as the reference standard. Group 2 included consecutive patients of the cardiovascular risk clinic, recruited in 2021, all of whom were at high or very high cardiovascular risk according to the European guidelines on cardiovascular disease prevention [18], as controls who likely had increases in IMT. The group 2 exclusion criteria included past or present signs or symptoms of GCA and an erythrocyte sedimentation rate of > 40 mm/h.

Patients and controls provided informed written consent upon entry into the study protocol. This study was approved by the Ethics Committee of the Hospital Universitario La Paz (HULP: PI-3040 and HULP: PI-3992). The medical history, clinical examination, laboratory data, and US were performed at the time of each subject’s enrolment in the study.

### Vascular US of the large vessel arteries

US examinations of the axillary, subclavian, and proximal and distal common carotid arteries (CCAs) were performed systematically using the Mylab X8 system (Esaote, Genoa, Italy, 2017). We used a 4–15-MHz probe with a gray scale frequency of 15 MHz and a Doppler frequency of 4.5 MHz, an adjusted color gain, and a PRF of 3000 Hz.

The CCA bifurcation and proximal portions of the internal and external carotid arteries on both sides were scanned for plaques using transverse and longitudinal views. A carotid plaque was defined as a focal structure that encroached into the arterial lumen by at least 0.5 mm or 50% of the surrounding IMT value or that demonstrated a thickness > 1.5 mm measured from the media-adventitia interface to the intima-lumen interface [19]. Carotid plaques were not considered as a halo sign, only diffuse non-focal homogeneous hypoechoic wall thickening was considered as a potential confounding factor with the halo sign associated with the diagnosis of GCA. IMT was measured in B mode, at the distal wall of the left and right CCAs, 10 mm proximal to the carotid bifurcation [19]. For analysis, the average of multiple measurements was used as IMT values recorded in either the right or left vessels. At the proximal CCA, we explored the CCA area distal of the clavicle shadow, and as proximal as US examination was possible, the IMT was measured at the distal wall.

**Fig. 1:**
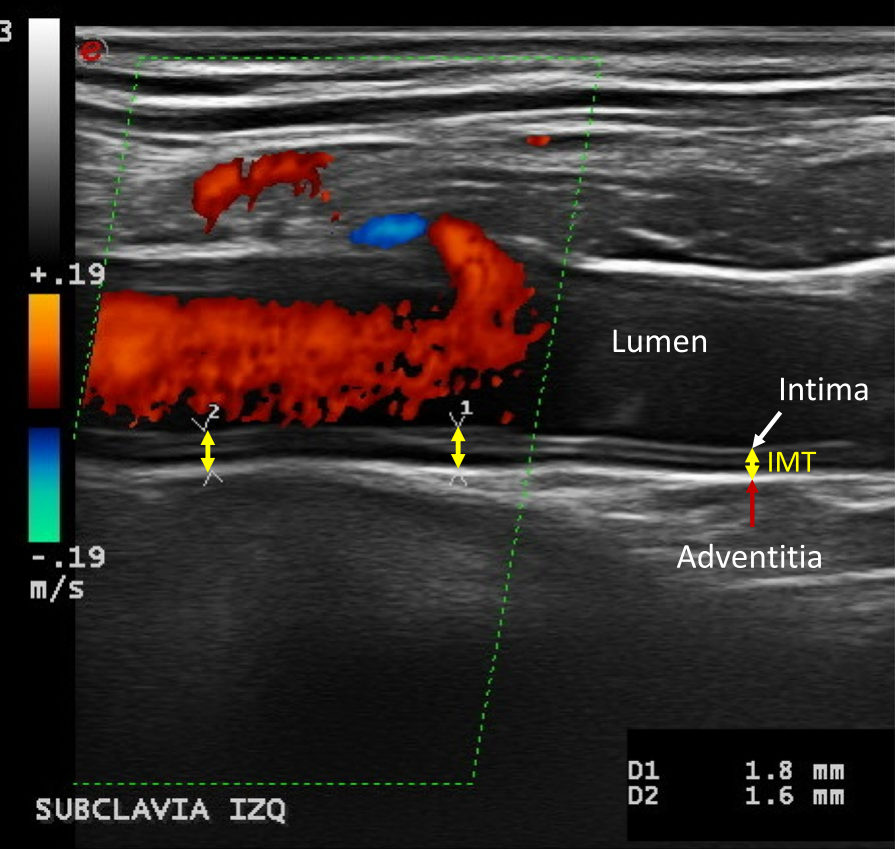
IMT measurement: intima-media thickness (IMT) is measured as the distance between lumen-intima (white arrow) and media-adventitia (red arrow) interfaces. Examples are shown in yellow arrows. In this case, we see an example of mid- to hyperechoic long segmental thickening of the intima-media displaying several visible lines with loss of the typical double line pattern in a longitudinal scan of the left subclavian artery, corresponding to the increased IMT with chronic changes

**Fig. 2:**
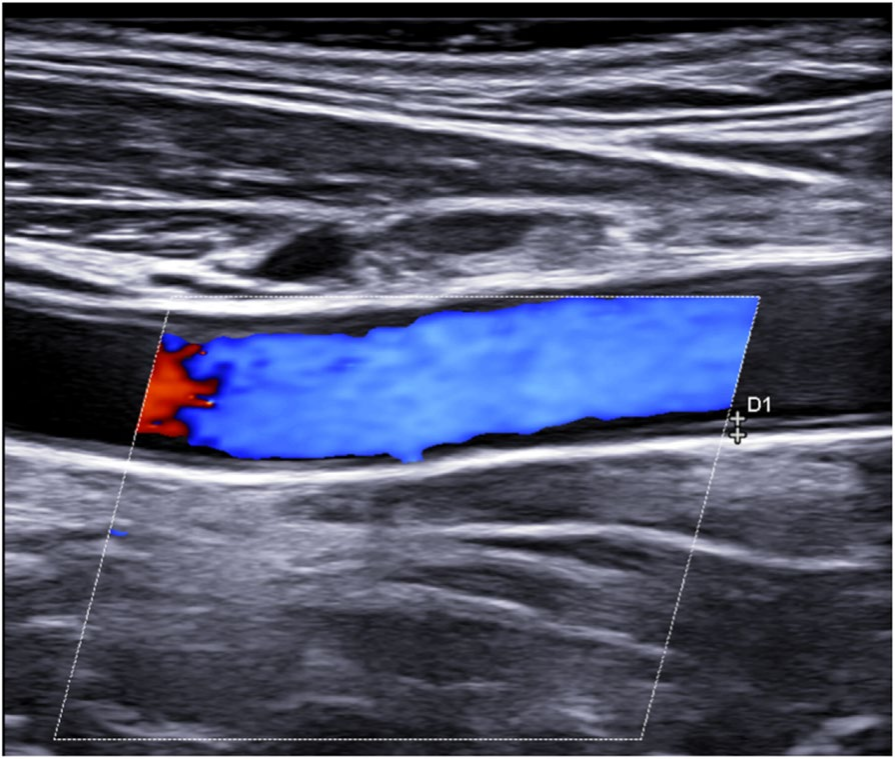
Longitudinal scan of a normal carotid artery

**Fig. 3:**
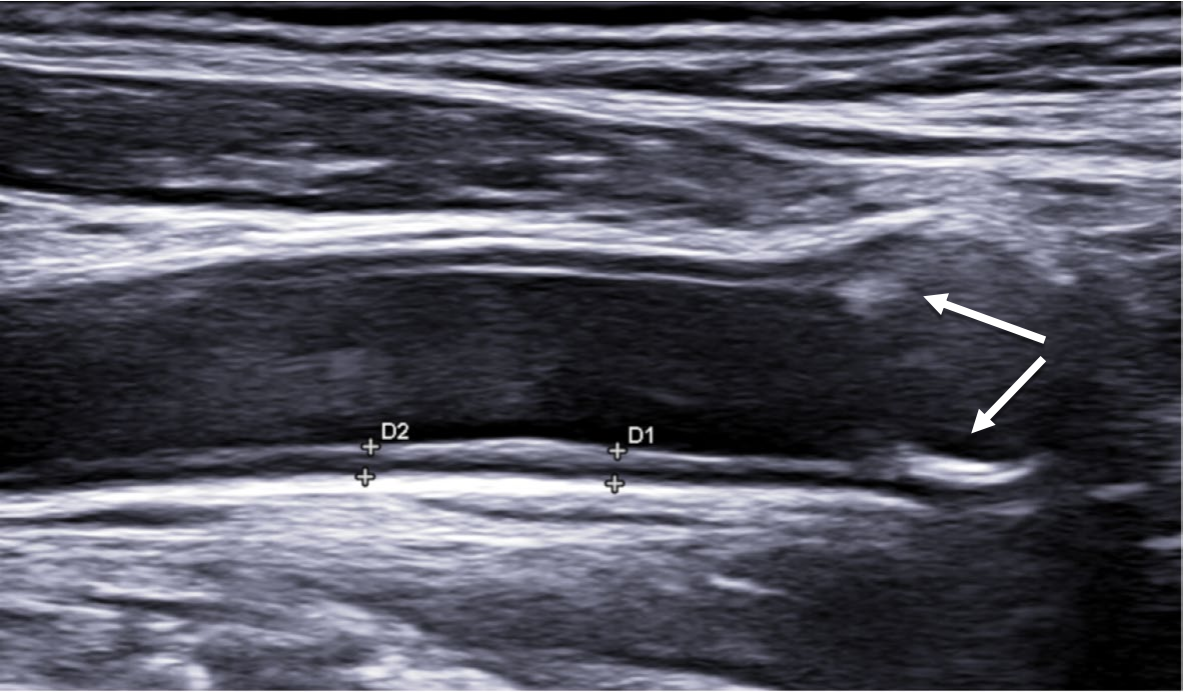
Longitudinal scan of a carotid artery with atherosclerosis: IMT 1.2 mm and atherosclerotic plaques (arrows)

**Fig. 4:**
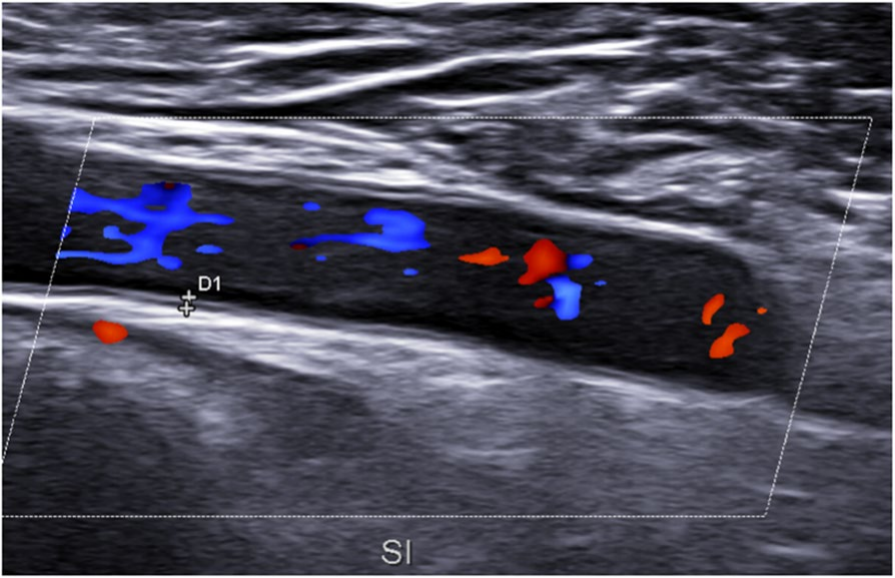
Longitudinal scan of a normal subclavian artery: IMT 0.45 mm

**Fig. 5:**
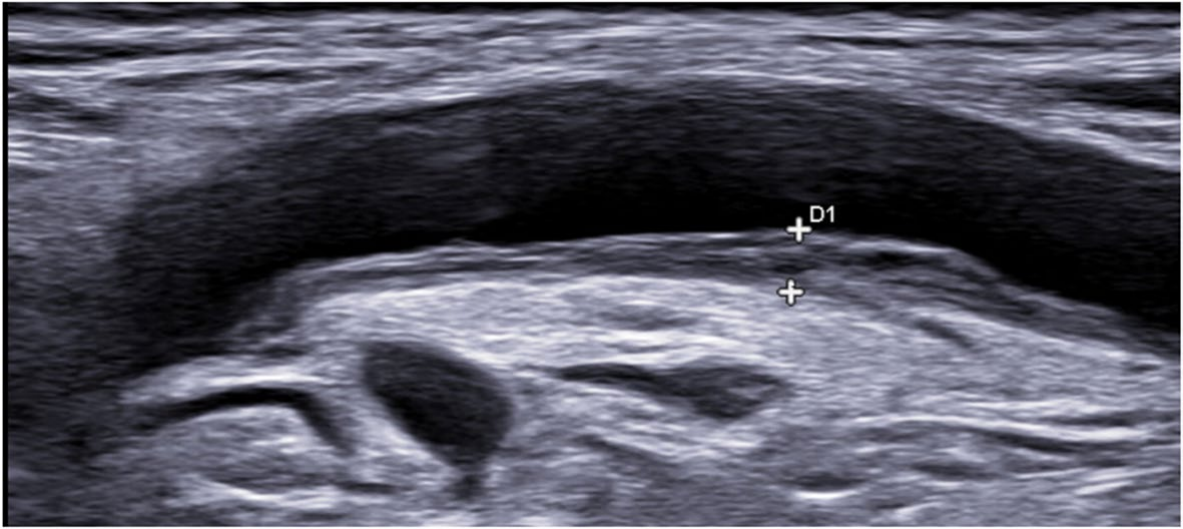
Longitudinal scan of axillary artery: chronic changes in the intima-media complex in GCA patient: IMT 2.3 mm

The OMERACT definitions of the halo sign and of normal arteries were applied [14]. The axillary and subclavian arteries were fully and bilaterally examined in the long and short planes. In each vascular territory, the thickness of the halo was measured at the point of maximum thickness in the longitudinal plane. For extra-cranial arteries, an IMT cutoff value of  ≥ 1.0 mm was considered positive in our hospital based on previous studies [15, 20–22]. Different composite IMT/halo scores were developed based on different quantitative values of the percentile measures of the different vessels and the number of vessels with halo thickness ≥ 1.0 mm. A schematic figure of IMT measurement and examples of US of normal arteries, atherosclerosis, and vasculitis in GCA patients are shown in Figs. 1, 2, 3, 4, and 5.

All the CDS examinations in our study were routinely performed by two experienced rheumatologists (IMH and EDM) with more than 5 and 15 years of experience in GCA US, both with excellent reliability. Five-second videos of every vessel were stored for reliability evaluation, and a consensus reader exercise about measures was performed on the videos several months after the initial examination. The ultrasonographers were not blinded to the clinical information and laboratory data of patients.

### Statistical analysis

The SPSS software (version 20.0; IBM, Armonk, NY, USA) was used for statistical analysis. Quantitative variables are presented as means with standard deviation, medians, and percentiles. The Kolmogórov-Smirnov test was used to check the distribution of the sample. Student’s *t*-test for independent samples was used to compare the continuous variables, and the Mann–Whitney *U* test was used for continuous nonparametric variables. Receiver operating characteristic (ROC) curve analysis was performed to estimate the LV IMT cutoff values. For interreader reliability purposes, we performed the kappa test on the recorded videos.

## Results

### Patient characteristics

During the recruitment time, 58 LV-GCA and 55 atherosclerosis patients were collected. To avoid bias, 14 GCA patients and 13 atherosclerosis patients were excluded to avoid significant differences in age and sex. The final analysis study included 44 patients with LV-GCA and 42 patients with atherosclerosis as controls. The LV-GCA group included 24 females and 20 males with a mean age of 72.8 ± 7.6 years. The atherosclerosis group included 25 males and 17 females with a mean age of 70.8 ± 6.5 years. The mean body mass index measured 27.1 ± 3.8 in the GCA group and 26.2 ± 2.4 in the atherosclerosis patients. There were no statistical differences between the groups in regard to age (*p* = 0.19), sex (*p* = 0.20), or body mass index (*p* = 0.32). Regarding other cardiovascular risk factors such as arterial hypertension (*p* < 0.01), diabetes (*p* < 0.01), dyslipidemia (*p* < 0.01), smoking (*p* < 0.01), and alcohol consumption (*p* < 0.01), all proved to be statistically more frequent in the atherosclerotic group, with frequencies of arterial hypertension (58% vs. 41%), diabetes (44% vs. 23%), dyslipidemia (76% vs. 51%), and ever smoking (44% vs. 18%), in atherosclerosis vs. LV-GCA patients.

#### Intima-media thickness

The IMT of the atherosclerotic and GCA vessels had a normal distribution. IMT values were significantly higher in all vessels of patients with GCA vs. atherosclerosis (Table 1). When we analyzed the different arteries, we found that only in the atherosclerotic group did the subclavian right artery (IMT 0.75 mm) achieve a significant difference with respect to the left side (IMT 0.68 mm). In the other arteries, there were no significant differences between the left and right sides, either in atherosclerosis or in GCA patients.

**Table 1:** IMT values of the carotid, axillary, and subclavian arteries

	**Carotid distal right**	**Carotid distal left**	**Subclavian right**	**Subclavian left**	**Axillary right**	**Axillary left**
Atherosclerosis patients (*n* = 42)						
Mean	0.89	0.88	0.75	0.68	0.64	0.66
Median	0.85	0.87	0.76	0.70	0.59	0.64
Standard deviation	0.20	0.21	0.19	0.16	0.20	0.16
Minimum	0.53	0.46	0.51	0.36	0.40	0.43
Maximum	1.40	1.70	1.60	1.10	1.50	1.00
Percentile 25	0.74	0.75	0.61	0.62	0.53	0.54
Percentile 50	0.85	0.87	0.76	0.70	0.59	0.64
Percentile 75	0.87	1.00	0.84	0.77	0.70	0.77
Percentile 90	0.95	1.10	0.92	0.87	0.82	0.89
GCA patients (*n* = 44)						
Mean	1.04	1.07	1.18	1.21	1.23	1.14
Median	1.00	1.00	1.15	1.20	1.20	1.15
Standard deviation	0.28	0.26	0.31	0.31	0.45	0.29
Minimum	0.55	0.60	0.60	0.67	0.46	0.57
Maximum	1.70	1.50	1.80	1.90	2.3	1.70
Percentile 25	0.86	0.87	0.90	1.00	0.95	0.93
Percentile 50	1.00	1.00	1.15	1.21	1.20	1.15
Percentile 75	1.15	1.30	1.40	1.32	1.45	1.40
Percentile 90	1.52	1.43	1.67	1.80	2.1	1.45
*P*-value of the mean measure atherosclerosis vs. GCA	0.01	0.001	0.001	0.001	0.001	0.001

#### Discriminant validity of the halo sign

Patients with GCA had significantly higher IMT mean values in all of the vessels explored than atherosclerosis patients, although 17 (38.6%) patients in the atherosclerosis group had an increased thickness (≥ 1 mm) in at least one explored vessel, which mimicked GCA. Most of the pathological findings were observed in the distal CCA near bifurcation: 14 (33.3%) cases. The proximal CCA was affected in 5 cases (11.9%), one case showed no other affected vessels. Subclavian affectation was present in 3 cases (7.1%) (two bilateral) while 2 cases (4.7%) had axillary artery affectation (one bilateral). This was a different pattern than that found in the GCA group, where axillary IMT ≥ 1 mm was found in 31 patients (70.5%), subclavian in 30 patients (68.2%), and CCA showed a halo sign in 28 (63.6%) patients at the distal area near the bifurcation, while 11 patients (25%) had a proximal increase. Slightly more than half of the patients in the GCA group (53%) had carotid atheromatous plaque. As per the study’s inclusion criteria, all patients in the GCA group had at least one vessel affected with the halo sign. The frequency and localization data are shown in Table 2.

**Table 2:** Number of arteries with intima-media thickness (IMT) ≥ 1 mm in GCA and atherosclerosis patients

	**GCA (*n* = 44)**		**Atherosclerosis (*n* = 42)**	
**Axillary**	Right	27	Right	2
	Left	23	Left	1
	Bilateral	19	Bilateral	1
	Any	31	Any	2
**Subclavian**	Right	20	Right	2
	Left	25	Left	1
	Bilateral	20	Bilateral	0
	Any	30	Any	3
**Common carotid distal**	Right	21	Right	8
	Left	19	Left	11
	Bilateral	12	Bilateral	5
	Any	28	Any	14
**Common carotid proximal**	Right	4	Right	3
	Left	9	Left	3
	Bilateral	2	Bilateral	1
	Any	11	Any	5

When we analyzed the results, we can see that the main differences between atherosclerosis and LV-GCA were the type of artery affected and the number or branches with an IMT or halo sign ≥ 1 mm. The presence of more than one arterial vessel affected between axillary and subclavian arteries (score of 4 vessels) or the presence of at least 3 in the carotid, axillary, and subclavian arterial examination (score of 6 vessels) achieved a 95% diagnostic accuracy for GCA with only 2 atherosclerosis patients misdiagnosed. The quantitative score based on ponderation of percentile measures in the different vessels did not achieve more precision and it was more time-consuming.

#### Reliability

The result of the interrater analysis between (E.D.M. and I.M.H.) revealed excellent reliability, with a kappa value of 0.842 (*p* < 0.01) for LV involvement and a kappa 0.865 (*p* < 0.01) for diagnosis of GCA.

## Discussion

The diagnosis of LVV, especially extra-cranial involvement in GCA is mainly based on imaging. In recent years, the use of US for this purpose has increased exponentially due to its availability, safety, tolerability, low cost, and because it can be done at the same time as the consultation or during the first visit to the fast-track clinic. Therefore, US has become the first imaging test to be performed in most cases.

The halo sign has been defined by OMERACT as representative of LV-GCA, and IMT measures of 0.9–1 mm have been used as cutoff points in the literature [15, 17, 20–22] but this cutoff has been not validated yet. IMT increases have also been reported and well-known in atherosclerosis cases, mainly in carotid bifurcation. However, carotid involvement is also frequently observed in patients with GCA in positron emission tomography with 18F-Fluorodeoxyglucose (18-FDG PET) [23, 24] and CT angiography studies [7]. Therefore, in some cases, the differential diagnosis between both diseases may be challenging.

We had previously reported a study that showed as cutoff point achieved sufficient accuracy to differentiate between the temporal affectation in GCA in front of increased in the thickness of these arteries in atherosclerosis patients [12]. In the present study, we evaluated the influence of atherosclerosis on extra-cranial arteries and explored whether it would be possible to perform an accurate differential diagnosis between atherosclerosis and inflammatory wall thickness in GCA as a principal confounder. Our results showed that all the vessels explored had a higher IMT in GCA than in atherosclerosis patients, but that a third of the latter showed IMT values and aspects that mimicked GCA in at least one vessel. It is important to note that our patients were recruited from a high-risk cardiovascular clinic. In this sense, our study is in agreement with one of the largest carotid IMT studies [25], even at the 97th percentile in those > 85 years of age, which was a lot smaller than the halo wall measurement in the present study. This supports the value of US in the differential diagnosis of GCA in front of one of the principal confounders as atherosclerotic disease.

Previous studies showed the “slope sign” as a specific US feature in the axillary arteries of patients with LV-GCA [21]. Unlike atherosclerosis, the thickening observed in vasculitis is smooth, homogenous, and continuous up to a transitional point, where the IMT gradually slopes downwards back to a normal level (< 1.0 mm). The authors therefore postulated that evaluation of this slope might help differentiate vasculitis from atherosclerosis [21]. While we agree with this observation by Dasgupta et al., we encountered some difficulties in clinical practice because we can see diffuse increased of wall thickness not only in a patched pattern which made it difficult the differential diagnosis. In this sense, we considered it relevant that, as shown in Table 2, only 2 patients in the atherosclerosis group showed IMT ≥ 1 mm in the axillary arteries and 3 in the subclavian arteries. Thus, if we use the cutoff point of more than one vessel affected in the score of subclavian and axillary arteries (score of 4 vessels), only 2 patients with atherosclerosis can be misclassified as GCA, one patient with axillary involvement and other with subclavian involvement. Carotid arteries are not so discriminating, and if we use the score of 6 vessels including carotids, we need at least 3 vessels affected to achieved a 95% of accuracy in the differential diagnosis between atherosclerosis and GCA. As far as we know, this have not been previously checked.

One limitation of this study was the small sample size included for each group. However, the differences achieved were statistically significant, and we think that future studies with larger samples will confirm our results. Another limitation is the influence of age and sex as other risk factors in the IMT measurement, possibilities that clinicians should be aware of in clinical practice. To minimize this, we selected patients paired by age and sex. An additional limitation was the impossibility of using paired samples for all the atherosclerotic risk factors, due to the fact that we would have needed a sample size 2–3 times larger if we hoped to match all of these cases. However, we believe that the unpaired factors would have increased the IMT in the group with vasculitis, which had a lower frequency of these risk factors. This, in any case, would have increased the differences and made the discriminant validity easier.

In summary, the patterns of arterial inflammation in LV-GCA can be quantified by US and show a differential pattern than those of atherosclerosis. We propose that in the assessment of LV, with an IMT cutoff value of  ≥ 1 mm, a simple halo count method of at least 1 affected vessel in the count of bilaterally axillary and subclavian arteries (score of four vessels), or at least 3 vessels in the count of six vessels (including CCA), shows a precision upper 95% in the diagnosis of GCA. The clinical application of these halo scores warrants further validation but opens a new challenge in the diagnosis of GCA in front of one of the principal confounders in the differential diagnosis.

## Data Availability

The data underlying this article will be shared on reasonable request to the corresponding author. All data relevant to the study are included in the article.

## Abbreviations

GCA: Giant cell arteritis
LV-GCA: Large vessel giant cell arteritis
US: Ultrasound
IMT: Intima-media thickness
CCAs: Common carotid arteries
CDS: Color Doppler sonography
EULAR: European Alliance of Associations for Rheumatology
OMERACT: Outcome Measures in Rheumatology Clinical Trials
ROC: Receiver operating characteristic
18-FDG PET: 18F-Fluorodeoxyglucose
